# High nuclear expression of STAT3 is associated with unfavorable prognosis in diffuse large B-cell lymphoma

**DOI:** 10.1186/1756-8722-4-31

**Published:** 2011-08-01

**Authors:** Wu ZL, Song YQ, Shi YF, J Zhu

**Affiliations:** 1 Department of Lymphoma, Peking University School of Oncology, Beijing Cancer Hospital & Institute; Key Laboratory of Carcinogenesis and Translational Research (Ministry of Education); Beijing 100142, China; 2 Department of Pathology, Peking University School of Oncology, Beijing Cancer Hospital & Institute; Key Laboratory of Carcinogenesis and Translational Research (Ministry of Education); Beijing 100142, China

## Abstract

**Background:**

The purpose of the study was to investigate the expression and prognostic value of STAT3 in diffuse large B-cell lymphoma (DLBCL).

**Methods:**

Seventy-four DLBCL patients from 2001 to 2007 were reviewed in the study. The STAT3 expression in their tumor tissues was examined using the immunohistochemistry (IHC) method, and evaluated for its association with clinicopathological parameters.

**Results:**

Strong nuclear staining of STAT3 and phosphorylated-STAT3^tyr705 ^(P-STAT3) were observed in 19 cases (25.7%) and 24 cases (32.4%), respectively, and the expression levels were highly consistent between them (*P *= 0.001). The high nuclear expression of STAT3 was more frequent in the non-germinal center B cell-like (non-GCB) DLBCL than that in the GCB subtype, but not reaching significance (*P *< 0.061). The high nuclear expression of STAT3 was found to be correlated with poor overall survival (OS) (*P *= 0.005). Multivariate Cox regression analysis showed that the STAT3 expression was an independent prognostic factor for DLBCL patients regardless of CHOP or R-CHOP regimen used as the first-line therapy.

**Conclusion:**

STAT3 is more frequently expressed in non-GCB DLBCL than that in GCB subtype, and its strong nuclear expression is correlated with poor OS in DLBCL.

## Introduction

Diffuse large B-cell lymphoma (DLBCL) is defined by the World Health Organization (WHO) Classification as a heterogeneous entity, encompassing morphologic and genetic variants, and variable clinical presentations and outcomes [1]. It accounts for 80% of aggressive lymphomas [2]. International Prognostic Index (IPI) is currently used to predict the prognosis in DLBC [3], but its role is limited[4]. Molecular subtypes of germinal center B cell-like (GCB) and non-germinal center B cell-like (non-GCB) DLBCL subtypes are proposed to stratify the prognosis of DLBCL in addition to the IPI score [5-7], but the application of Rituximab reduced the prognostic difference between the two subtypes [8,9]. More prognostic markers should be identified for DLBCL.

The Signal Transducers and Activators of Transcription (STAT) family members play important roles in transcriptional regulation and signal transduction, in which STAT3 plays a critical role in regulation of cell proliferation and survival [10] and is a critical transcription activator in angiogenesis [11]. Hypermethylation silencing of SOCS (the Suppressor of Cytokine Signaling) genes leads to reactivation of STAT pathway, resulting in the resistance to ABT-869, a promising multi-targeted tyrosine kinase inhibitor [12]. STAT pathway also triggers the activity of receptor-associated Janus kinase (JAK) family members and cross-talks with the nuclear factor-κB (NF-κB) pathway, which is an important molecular pathogenesis of lymphoma [13]. Thus the STAT family has been actively studied as one of molecular targets for anti-neoplastic therapy [14].

Expression of STAT3 in DLBCL subtypes may be variable according to *in vitro *studies [15,16]. The cell line studies showed that the activated B cell-like (ABC) DLBCL had the highest level of STAT3 mRNA, roughly 2-fold higher than that in the GCB DLBCL[15,16]. However, the STAT3 expression and its prognostic value in different subtypes of DLBCL tumors were not investigated. In the study, we investigated the expression level and frequency of STAT3 in DLBCL tumors, the difference of STAT3 expression in different DLBCL subtypes, and its prognostic value in DLBCL patients.

## Materials and methods

### Patients

Seventy-four consented patients with DLBCL in the Beijing Cancer hospital from 2001-2007 were studied. In 58 patients, 27 cases were treated with R-CHOP and 31 cases with CHOP as first-line regimens. The clinical research protocol was approved by our Institutional Review Board (IRB). Archived formalin-fixed and paraffin-embedded tumor tissues were obtained from our Department of Pathology.

### Immunohistochemical analysis (IHC)

4 μm thick sections were mounted on APES-coated slides. After dewaxing in xylene and rehydrating in a gradient concentration of ethanol, the slides were immersed in methanol containing 0.3% hydrogen peroxide for 15 minutes to block endogenous peroxidase activity. All slides were pretreated with an antigen retrieval method by heating the slides in an autoclave in citrate buffer (10 mM, pH 6.0) for 90 seconds except those stained for P-STAT3. EDTA-Tris buffer (1 mM, pH 9.0) was used for pretreating before P-STAT3 staining. After rinsing in TBS (pH7.6), the specimens were incubated for 2 h at 37°C with anti-STAT3 antibody (sc-7179 rabbit polyclonal antibody, Santa Cruz Biotechnology) for STAT3, anti-P-STAT3 antibody (9145, rabbit monoclonal antibody, Cell Signaling Technology) for P-STAT3^Tyr705^, and antibodies for BCL6, CD10, MUM-1 (Santa Cruz Biotechnology). Subsequently, all slides were incubated with Envision HRP antibody working fluid (Dako Company) for 30 minutes at 37°C, and then developed with DAB-H_2_O_2 _solution (Dako Company). The cell nuclei were stained with Meyer's hemotoxylin. The normal tonsil tissue was used as a negative control and breast cancer tissue stained positive was used as a positive control for STAT3 and P-STAT3 in all experiments. For technical details, see the manufacturer's instructions for each reagent.

IHC staining was evaluated by two independent experienced pathologists, who were blinded to the clinical data. As for the nuclear staining, at least 100 tumor cells per specimen were counted and only specimens showing moderate to strong immunoreactivity were considered positive. Staining was considered strong positive when > 75% of tumor cell nuclei were stained positive for STAT3 and > 30% of tumor cell nuclei for P-STAT3. Specimens stained positive for STAT3 ≤ 75% and ≤ 30% for P-STAT3 were considered weak immunoreactivity.

### Statistics

The Chi-square test was used to analyze the consistence of expressions of STAT3 in nucleus and P-STAT3. Correlation analysis of the STAT3 expression and the P-STAT3 level with clinicopathological variables was performed by two-sided Chi-square test. Kaplan-Meier method was used to estimate difference of OS. OS was defined as the time from diagnosis to death or the last follow-up. The Cox regression model was used to evaluate the prognostic value. The statistical software SPSS16.0 was used for all the statistical analysis.

## Results

### Patient characteristics

All patients had complete follow-up information from the Tumor Registry Office in our hospital. The clinicopathological characteristics are summarized in Table 1. Fifty five patients were younger than 60 years old. Male and female patients were 30 and 44, separately. Twenty nine patients were diagnosed with B symptoms, 50 patients had stage III-IV diseases and 50 patients were diagnosed with the non-GCB subtype.

**Table 1:** Clinicopathological parameters and their correlations with STAT3 nuclear expression

Clinical Parameters	*No.#*	Nuclear Staining		*P Value*	Clinical Parameters	*No.#*	Nuclear Staining		*P Value*
		Low positive	Strong positive				Low positive	Strong positive	
Gender					Stage				
Male	30	20	10	0.165	I~II	24	18	6	0.582
Female	44	35	9		III~IV	50	37	13	
Age					IPI				
< 60	55	43	12	0.161	0~2	48	39	9	0.099
≥ 60	19	12	7		3~4	22	14	8	
B symptoms					Bulky mass				
positive	29	23	6	0.306	≥ 10 cm	9	6	3	0.440
negative	45	32	13		< 10 cm	55	47	16	
LDH					Molecular subtypes				
positive	28	19	9	0.235	GCB	24	21	3	0.061
negative	46	36	10		non-GCB	50	34	16	
β2-MG					Treatment regimens				
positive	46	34	12	0.513	CHOP	31	22	9	
negative	17	12	5		R-CHOP	27	19	8	
ESR									
positive	38	28	10	0.406					
negative	29	23	6						

### STAT3 expression

**Figure 1:**
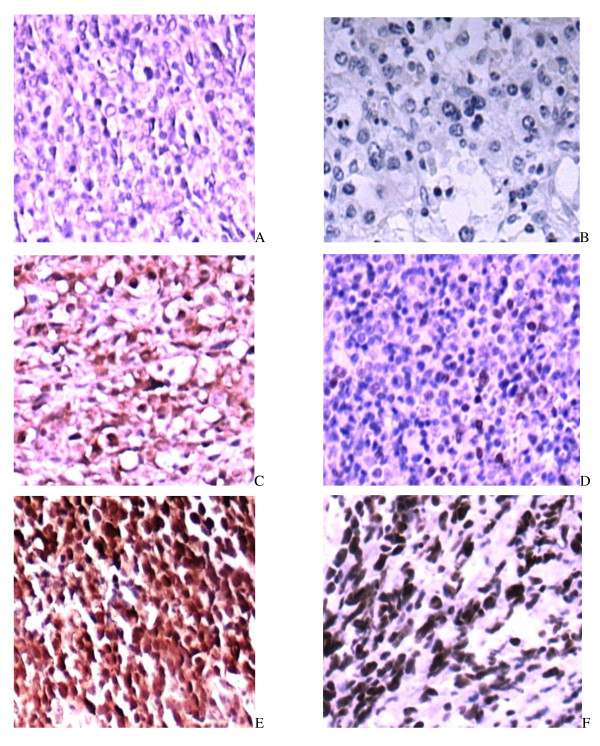
**STAT3 expression and P-STAT3 level in DLBCL (A) negative nuclear staining of STAT3, (B) negative nuclear staining of P-STAT3, (C) weak nuclear staining of STAT3, (D) weak nuclear staining of P-STAT3, (E) strong nuclear staining of STAT3, (F) strong nuclear staining of P-STAT3**.

Among the 74 patients, 66 cases (89.19%) had the STAT3 expression, including 19 cases (25.7%) with strong nuclear staining of STAT3, and 24 cases (32.4%) with strong nuclear staining of P-STAT3. Representative staining outcomes were shown in Figure 1. There existed a consistence between the STAT3 expression and the P-STAT3 level (*P *= 0.001), indicating the reliability and accuracy of our IHC analysis (Table 2).

**Table 2:** Relationship between the STAT3 expression and the P-STAT3 level

		STAT3 expression in nucleus		Total	*P Value*
		Weak positive	Strong positive		
**P-STAT3**	Low positive	43	7	50	
	Strong positive	12	12	24	0.001
**Total**		55	19	74	

### Correlation between the nuclear expression of STAT3 and clinicopathological parameters

We observed the associations of the STAT3 nuclear expression with IPI score and molecular subtypes, but no statistical significances were reached (*P *= 0.099 and *P *= 0.061, respectively). No association was found between the STAT3 nuclear expression and other factors, including B symptoms, age of onset, clinical stage, and erythrocyte sedimentation rate (ESR), lactate dehydrogenase (LDH), and tumor size (Table 1).

### Association between the nuclear expression of STAT3 and overall survival

**Figure 2:**
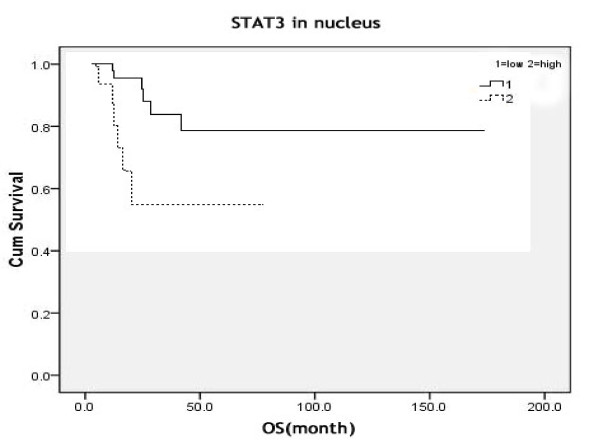
**Kaplan-Meier curve of overall survival (OS) using STAT3 nuclear expression**.

Kaplan-Meier analysis showed that strong STAT3 nuclear expression was correlated with poorer OS (*P *= 0.005) (Figure 2). Other factors such as serum LDH level, clinical stage, B symptoms, tumor size, and IPI score were also shown to be correlated with OS (data not shown) as reported in other studies, which confirmed our data is reliable. A forward stepwise multivariate Cox model analysis, incorporating the above factors, demonstrated that the nuclear expression of STAT3 (*P *= 0.001), LDH level (*P *= 0.002) and tumor size (*P *= 0.025) were independent prognostic factor for OS.

**Figure 3:**
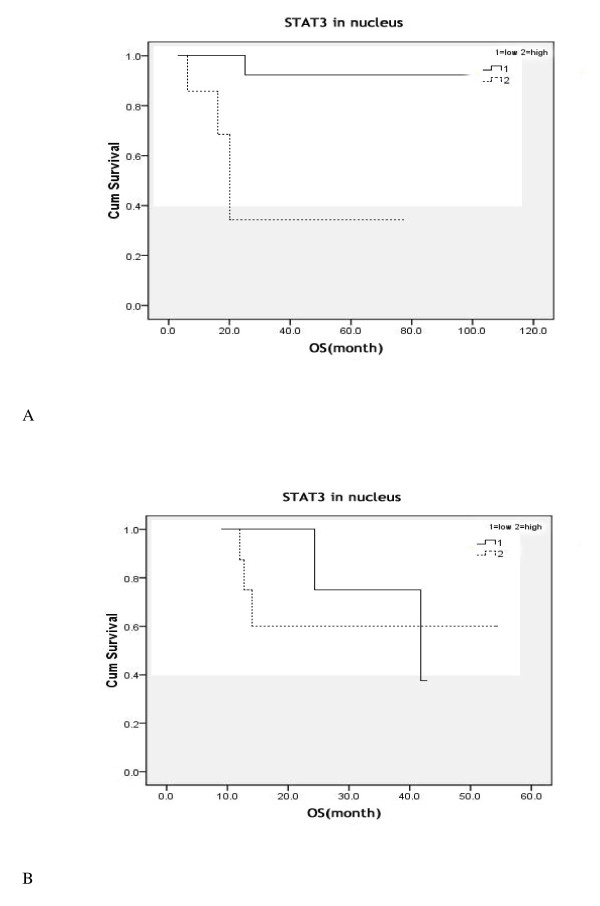
**Kaplan-Meier curve of overall survival (OS) in patients with different treatment regimens using STAT3 nuclear expression**. (A) Patients received the CHOP regimen; (B) Patients received the R-CHOP regimen.

To analyze the prognostic implication of STAT3 in term of Rituximab therapy, we stratified all patients into two subgroups, the CHOP subgroup and the R-CHOP subgroup. In CHOP subgroup, high nuclear expression of STAT3 predicted poor survival (*P *= 0.001). In R-CHOP subgroup, 2 of 19 cases died of DLBCL in low STAT3 cohort and 3 of 8 cases died in high STAT3 cohort. No significant association was observed between the expression of STAT3 and prognosis (*P *= 0.216) in the R-CHOP subgroup. But the survival curve showed that high STAT3 expression indicated poor OS in the first 40 months. Thus, it needs to increase the sample size to confirm this result (Table 3, Figure 3).

**Table 3:** Correlation of STAT3 nuclear expression with overall survival

treatment	STAT3	OS		*P*
			
		positive	negative	
CHOP	Low	1	21	0.001
	High	3	6	
R-CHOP	Low	2	17	0.216
	High	3	5	

## Discussion

Lam LT[15] et al. reported that activated B-cell diffuse large B-cell lymphoma (ABC-DLBCL) had higher level of STAT3 mRNA than that in GCB-DLBCL. Detection with immunohistochemistry [15] showed that slightly more cases with high nuclear expression of STAT3 were observed in the non-GCB DLBCL group and the high expression rates were 12.5% and 32.4% in GCB and non-GCB subtypes, respectively. However, no statistical significance was found. This is most likely due to the small sample size. Our study showed that the frequency of high nuclear expression of STAT3 in DLBCL was 25.7% with 12.5% in GCB subgroup and 32% in non-GCB subgroup, but not reaching significance (*P *= 0.061).

Lam LT et al. [15] also demonstrated that high STAT3 expression in ABC-DLBCL patients correlated with inferior overall survival, but not with GCB-DLBCL patients. However, STAT3-high and STAT3-low subsets within ABC-DLBCL did not differ in prediction of overall survival. Our study showed that high nuclear expression of STAT3 in DLBCL possibly correlated with poor overall survival, especially in patients receiving CHOP regimen. This poor outcome may be explained at least in part by the multiple cellular functions of STAT3, which is a critical component of diverse signal transduction pathways[15,17,18]. STAT3 regulates the expression of a number of genes (e.g. survivin, bcl-xl, mcl-1) that modulate cell survival, differentiation, and proliferation (e.g. c-myc, cyclin D1, p21, cyclin E), invasion and metastasis (e.g. matrix metalloproteinase-9 and 2)[19], and angiogenesis (e.g. vascular endothelial growth factor) [11,20,21]. STAT3 can restrain anti-tumour immune responses [22-27] and regulate key cancer-promoting inflammatory mediators, which can initiate or promote oncogenic transformation, and genetic and epigenetic changes in malignant cells [28,29].

Our study also demonstrated the possibility of using immunohistochemistry to detect STAT3 expression in routine pathologic specimens, which may enable us conveniently to identify DLBCL cases with poor clinical outcome, and subsequently guides us to adopt more intensive treatment for those patients.

Since STAT3 plays a critical role in tumor initiation and progression, inhibition of STAT3 activation would be an effective approach for cancer prevention and treatment. Our findings may provide a basis for the application of STAT3 inhibitors in the future.

## Grant Support

This study was supported by the grant of the National Science Foundation Committee (NSFC) of China (No. 30973484)

## Conflicts of interests

The authors declare that they have no competing interests.

## Authors' contributions

ZJ designed the study and reviewed the final manuscript; WZL collected and analyzed data, and drafted the manuscript; SYQ participated in the study design and helped draft the manuscript and reviewed the final manuscript; SYF helped the IHC staining. All authors read and approved the final manuscript.
